# Chronic pediatric diseases and risk for reading difficulties: a narrative review with recommendations

**DOI:** 10.1038/s41390-022-01934-y

**Published:** 2022-02-04

**Authors:** Donna Perazzo, Ryan Moore, Nadine A. Kasparian, Megan Rodts, Tzipi Horowitz-Kraus, Lori Crosby, Brian Turpin, Andrew F. Beck, John Hutton

**Affiliations:** 1 Reading and Literacy Discovery Center, Cincinnati Children’s Hospital Medical Center and Department of Pediatrics, University of Cincinnati College of Medicine, Cincinnati, OH USA; 2 Division of General and Community Pediatrics, Cincinnati Children’s Hospital Medical Center and Department of Pediatrics, University of Cincinnati College of Medicine, Cincinnati, OH USA; 3 Division of Hospital Medicine, Cincinnati Children’s Hospital Medical Center and Department of Pediatrics, University of Cincinnati College of Medicine, Cincinnati, OH USA; 4 The Heart Institute, Cincinnati Children’s Hospital Medical Center and Department of Pediatrics, University of Cincinnati College of Medicine, Cincinnati, OH USA; 5 Center for Heart Disease and Mental Health, Heart Institute and Division of Behavioral Medicine and Clinical Psychology, Cincinnati Children’s Hospital Medical Center and Department of Pediatrics, University of Cincinnati College of Medicine, Cincinnati, OH USA; 6 Center for Clinical and Translational Science and Training and Division of Behavioral Medicine and Clinical Psychology, Cincinnati Children’s Hospital Medical Center and Department of Pediatrics, University of Cincinnati College of Medicine, Cincinnati, OH USA; 7 Division of Oncology, Cincinnati Children’s Hospital Medical Center and Department of Pediatrics, University of Cincinnati College of Medicine, Cincinnati, OH USA; 8 Educational Neuroimaging Center, Faculty of Education in Science and Technology and Faculty of Biomedical Engineering, Technion, Haifa, Israel

## Abstract

**Abstract:**

Literacy is a major social determinant of health, rooted in skills that develop during early childhood. Children arriving at kindergarten unprepared to learn to read are more likely to have low reading proficiency thereafter. General and health literacy are highly correlated, affecting understanding of health conditions, treatment adherence, and transition to self-care and adult healthcare services. The American Academy of Pediatrics (AAP) recommends literacy and school readiness promotion during well-visits and neurodevelopmental surveillance is emphasized across primary and subspecialty care. While genetic and environmental risk factors for reading difficulties are well-established, risks related to complex and chronic medical conditions are less appreciated and under-researched. This review applies an eco-bio-developmental framework to explore literacy across five complex chronic conditions affecting millions of children worldwide: asthma, cancer, congenital heart disease, epilepsy, and sickle cell disease. In each, integration of an efficient reading brain network may be impacted by direct factors, such as ischemia, anesthesia, and/or medications, and also indirect factors, such as altered parent–child routines, hospital stays, and missed school. By integrating literacy into care management plans for affected children, pediatric primary care and specialty providers are poised to identify risks early, target guidance and interventions, and improve academic and health outcomes.

**Impact:**

- While genetic and environmental risk factors for reading difficulties are well-established, risks related to complex and/or chronic medical conditions such as asthma, cancer, congenital heart disease, epilepsy, and sickle cell disease are substantial, less appreciated, and under-researched.
- General and health literacy are highly correlated, with implications for the understanding one’s health condition, treatment adherence, and transitioning to self-care, which is especially important for children with complex and/or chronic illness.
- Pediatric primary care and specialty providers are poised to integrate reading and literacy into care management plans for children with complex and/or chronic illness, including early screening, guidance, support, and interventions.

## Introduction

Literacy is a major social determinant of health and plays a prominent role in the lives of children and families across demographic spectra.^1^ Component skills emerge beginning in infancy via an integrative, neurodevelopmental process influenced by genetic, environmental, and medical risk factors.^2^ This process is well-suited for an eco-bio-developmental framework as advocated by the American Academy of Pediatrics (AAP) and recently proposed in a review by Hutton, et al.^3^ Here, a child’s home and other experiences (eco), genetic makeup, and medical concerns (bio) exert combined, sustained influence on reading development and subsequent academic, social and health outcomes. This framework can help optimize approaches to screening, guidance, and interventions beginning as early as possible.^4^

Low literacy levels are associated with numerous adverse outcomes, including aggressive and risk-taking behaviors, mood disorders, substance abuse, unintended pregnancy, crime, limited vocational potential, and poverty.^5–7^ Morbidity and financial costs accrue on individual, familial, and societal levels,^5^ estimated at $350 billion in the United States and $1.3 trillion worldwide.^8^ General and health literacy are also highly correlated, with implications for capacities to care for self and others.^9^ This is critical in terms of parenting, adherence to medical regimens, and transition planning, when young people with complex chronic health conditions learn to manage their own health care needs into adulthood.^10–12^

The AAP recommends a central role for pediatric providers in developmental surveillance, literacy promotion, and improving school readiness.^13^ Among the most straightforward and potentially impactful guidance is that parents read with their children (shared reading) as often and interactively as possible, beginning in infancy.^13,14^ Decades of research have established cognitive, social-emotional, and academic benefits of shared reading, including for children from at-risk backgrounds, such as those who experience trauma or live in poverty.^14–17^ Neuroimaging studies have also begun to quantify the benefits of shared reading during early childhood on brain structure and function.^18–20^ As there is no hardwired “reading network” in the brain, emergent literacy development requires integration of networks supporting a range of cognitive abilities (i.e., language, attention, visual, executive functions) through exposure and practice,^21^ ideally during early childhood when plasticity is high.^4^ Dysmaturation, disruption, or under-stimulation of neural circuits involved in this integrative process is the basis of reading difficulties.^22^ Excessive screen-based media use (screen time) has the potential to displace shared reading and other interactions with caregivers, further impacting developmental trajectories.^23^ Thus, the AAP recommends screen limits for all ages, while encouraging nurturing alternatives.^24^

Reading difficulties are not restricted to children with genetic risk factors (e.g., dyslexia) or in the context of an impoverished home literacy environment (HLE), which are each well-established.^25,26^ Pediatric medical conditions, particularly those that are chronic or complex, can also confer substantial risk. While reasonably expected with pervasive developmental disorders such as Trisomy-21 and severe autism,^27^ reading difficulties can also stem from deficits accompanying other conditions that are less straightforward. Potential mechanisms include direct effects on neural circuits in the emerging reading network, such as via neurovascular ischemia or insults, toxin exposure (e.g., chemotherapy), or neurodevelopmental structural differences.^21^ Indirect mechanisms include disrupted home and/or school routines by frequent medical appointments, disease flares, and/or prolonged hospital stays, particularly during early childhood.^28–31^ Extended time in the hospital and family stress^32^ may also negatively affect HLE via displaced shared reading time and/or competing priorities (i.e., diminished importance relative to physical health, rehabilitation and survival). Children with chronic illness are also prone to high levels of screen use, as they are more often sick and/or isolated at home or in the hospital.

The combined impact of these factors in the context of medical complexity and how they may be optimally managed are not well understood, reflecting major clinical and research gaps. For example, efficient tools to screen early literacy skills and risk factors have been validated,^33–35^ yet are not widely used in pediatric practice. Further, while evidence-based programs designed to enhance HLE are well-established in primary care (e.g., Reach Out and Read; ROR^36^) and shared reading is increasingly advocated in Neonatal Intensive Care Units (NICUs),^37,38^ no similar program exists during pediatric specialty or inpatient care.

While there are excellent reviews involving literacy development in “typical” children,^39^ those with genetic risk factors^40^ and for select conditions (e.g., hearing loss,^41^ prematurity,^42^) to our knowledge this has not been previously done in the context of chronic pediatric diseases. Among the most prevalent are asthma, cancer, congenital heart disease (CHD), epilepsy, and sickle cell disease (SCD),^43^ which combined, affect over 6-million children in the US (Table 1).^44–48^ The purpose of this review is to summarize evidence regarding cognitive development in young children with these conditions as it relates to reading and literacy within an eco-bio-developmental framework, including recommendations for pediatric providers and areas in need of research.

**Table 1:** Summary of prevalence, usual age of diagnosis, and associations and mechanisms of potential reading difficulties.

Disease	Prevalence (US)	Usual age of diagnosis	Links to reading difficulties	Potential mechanism
Asthma	7.5% (5.5 million)	2–5	Impairments in verbal memory, reading readiness, reading abilities, academic achievement	Steroid therapy, hospital admissions, sleep-disordered breathing, comorbidities (e.g., anxiety), disruption to home and school routines (e.g., absenteeism)
Cancer	15,780 new cases/year	Mean 5 years (0–14)	Impairments in attention, working memory, learning, and pre-academic skills	Chemotherapy/radiation, anesthesia, hospital admissions, disruption to home and school routines
CHD	1% (~1 million)	<1 year	Impairments in attention, working memory, language acquisition, kindergarten readiness	Anesthesia, cardiac bypass, hypoxia-ischemia, neurodevelopmental differences, hospital admissions, disruption to home and school routines
Epilepsy	(~470,000)	<10 years	Impairments in language, cognitive development, school readiness, attention, memory, speed of processing, executive functions, comprehension	Neurodevelopmental differences, anti-epileptic drugs, surgeries, comorbidities (e.g., ADHD, anxious and/or depressed mood), disruption to home and school routines
SCD	0.1% (~100,000)	<1 year	Impairments in cognitive development, executive functions, language processing	Stroke, silent cerebral infarct, ischemia, comorbidities (e.g., anxious/depressed mood), disruption to home and school routines

## Methods

Medical conditions were chosen based on four criteria: (1) prevalence in children, (2) typical age of diagnosis before age 6, when formal reading instruction commonly begins, (3) not caused by an isolated neurological insult (e.g., head trauma), and (4) not part of a broader developmental delay or genetic syndrome (e.g., autism, Trisomy-21). While clearly associated with reading difficulties, extreme prematurity and hearing loss were excluded, as the former is not a distinct diagnosis and is associated with heterogeneous health implications^49^ and the latter has been well-covered in scientific literature.^50^

Searches were performed in PubMed and Google Scholar using a thematic approach. These were conducted by specialists in their respective area, in collaboration with the lead and senior authors. Search terms included each medical condition plus relevant keywords (“literacy,” “emergent literacy,” “reading,” “reading difficulties,” “reading delay,” “cognitive,” “language,” “kindergarten,” “readiness,” “school,” “health literacy,” “transitioning”). Additional inclusion criteria were: (1) study population involved children under the age of 18 years, (2) the article was published in English, (3) peer-reviewed format, (4) could be obtained via journal access from the host institutions (CCHMC and the University of Cincinnati), and (5) published later than 1990. Qualitative, quantitative, and meta-analytic designs were allowed. Final decision to include was by unanimous agreement between respective specialists and the senior author, particularly in terms of relevance to reading and literacy outcomes.

### Narrative synthesis and interpretation of findings

#### Asthma

Asthma is the most common chronic childhood illness in the US, affecting as many as 5.5 million (7.5%) children under age 18.^51^ Symptoms often emerge between ages 2 and 3 years, but diagnostic criteria are most reliable after age 5.^52^ Asthma is most prevalent in children of minority race and ethnicity (e.g., Puerto Rican, Black) and in boys (12% vs. 8% girls).^53,54^ In general, severity skews highest in children from low-socioeconomic status (SES) backgrounds living in urban areas,^55,56^ related to greater exposure to air pollution (also linked to lower cognitive and brain development),^57^ unsanitary living conditions,^58^ and other triggers.^59^ As children from low-SES households face considerable disparities in kindergarten reading readiness and school outcomes,^60^ attributable in large part to less stimulating HLE and also less access to quality early childhood education,^61^ it is not surprising that these are amplified by an asthma diagnosis. For example, an age-matched study of asthmatic versus non-asthmatic children starting kindergarten in an urban school district found that asthmatics had significantly lower reading readiness skills.^62^ Asthma diagnosis has been identified as an independent, persistent predictor of low academic achievement, adjusting for covariates including gender and SES.^63^ Higher asthma severity (particularly hospital admission) has been associated with lower school readiness, reading abilities^64,65^ and academic performance, including in a large birth cohort study.^66^ A substantial mediator is school absenteeism, which is disproportionately higher in asthmatic children^67–69^ especially in urban areas.^70^ Inequitable reading and school outcomes for asthmatic children tend to persist or worsen over time, suggesting that those who start at a disadvantage tend to fall even farther behind.^71^

While missed school is clearly a driver of adverse reading outcomes in asthmatic children, evidence is inconclusive as to whether this is the sole or even major cause.^64,72–74^ Therapies may exert direct impacts, suggested by links between frequent exposure to oral steroids during acute illness and lower cognitive (memory) and school performance.^75,76^ Steroid use, especially when prolonged or frequent, has also been linked to emotional and behavioral difficulties,^77^ which in turn may affect school performance. However, studies have also suggested cognitive and academic benefits of steroid therapy, and so to what degree these confer or mitigate risk is unclear.^78^ Lower-quality sleep fueled by sleep-disordered breathing (SDB) is also a likely contributor to the lower reading and school performance, given the importance of sleep for healthy cognitive function and development.^79^ Higher rates of anxiety and household stress in asthmatic children likely exert indirect effects, negatively impacting performance.^80,81^

Lower reading abilities in asthmatic children are linked to a worse understanding of the illness, triggers, and actions needed to stay healthy (i.e., child health literacy).^82,83^ This is a major concern for children from low-SES backgrounds, who are often expected to manage their own medications and asthma action plans.^84,85^ In turn, lower health literacy is associated with poor adherence to treatment plans, increased medication errors, and more frequent and prolonged hospital admissions.^86,87^ Lower literacy abilities also likely impede the transition to self-care management in adolescence and into adulthood, worsening outcomes.^63^ While there have been intensive efforts to simplify care plans such as through picture-based educational materials,^88,89^ challenges related to general reading and literacy abilities remain.

Despite increasing evidence of associations between asthma, risk for reading difficulties, and low school achievement, little research has been conducted evaluating reading-related interventions or programs specifically for asthmatic children. One pilot program utilized a Saturday-school format to combine asthma education for 6–14-year-old children with moderate-severe asthma with instruction focused on reading accuracy, comprehension, and oral and written language skills.^90^ This involved weekly, three-hour sessions for 6 months and resulted in 22% fewer hospital admissions and 33% fewer emergency department (ED) visits. Benefits were highest for participants who showed improvement on a standardized reading comprehension battery (Grey Oral Reading Test; GORT), adjusting for age, sex, and ethnicity. Higher self-efficacy (Drew Self-Efficacy Scale), proposed to be fueled by higher literacy levels, was also related to fewer ED visits and hospitalizations.^90^ While challenging to implement, such “hybrid” interventions, providing support for both general and health literacy, show promise in terms of reduced morbidity and medical costs. Research is clearly needed to better understand the links between asthma and literacy, and to target interventions as early as possible.

#### Cancer

While rare overall, pediatric cancer diagnoses frequently present during early childhood (32% of cases between age 0 and 4 years).^91^ In the US, rates vary by region but are greatest in metropolitan areas, with approximately 11,000 children and adolescents under age 15 receiving a cancer diagnosis each year.^46^ Most common are leukemias and lymphomas (41.1%), central nervous system (CNS) tumors (25.4%), and sarcomas (11%).^92^ While cases have risen in past decades, so have five-year survival rates, from 58% in 1973 to 80% in 2019.^93^ Thus, there are currently over 420,000 adult survivors of childhood cancer in the US. Longevity is associated with the emergence of comorbidities, with an average of 4.7 chronic conditions by age 50.^94,95^ Most are linked to prolonged treatments involving surgery, CNS-directed radiation, and chemotherapy, which involve hospitalizations and frequent follow-up with specialists.^33^ These multimodal therapies (often involving CNS toxicity), along with cardiac, pulmonary, and endocrine system toxic mediators, coupled with long hospital stays and perturbed home environments, can affect neurocognitive development and fuel adverse reading and academic outcomes in an insidious manner.^96^ Late cognitive effects are especially prevalent, impacting 35% to 60% of childhood cancer survivors.^97,98^ For example, an analysis of outcomes data for 4- to 5-year-olds with brain tumors, non-CNS solid tumors, or leukemia referred for psychological evaluation found significant deficits in pre-reading skills (e.g., color, shape, letter, and number naming) compared to age-matched norms.^96^

The most common cancers in childhood are often heavily treated with CNS-directed therapies. Notably, intrathecal chemotherapy is integral to the treatment of leukemia, yet it can leave survivors with measurable gray matter changes and decline in memory performance and attentional capacity,^99,100^ core skills for reading comprehension.^2^ A large longitudinal study of acute lymphoblastic leukemia (ALL) survivors aged 1–18 years at diagnosis assessed neuropsychological functioning at three time-points over the two years post-chemotherapy treatment.^101^ Compared with unaffected siblings, chemotherapy-treated children had significantly higher rates of attention deficits, learning problems, and need for special education.^101^ While neurocognitive functioning improved over time, there were persistent attention and learning difficulties reported by caregivers, underscoring the need for sustained academic support.

CNS tumors are relatively common in children and represent a leading cause of cancer-related morbidity over time. Surgical resection is a nearly invariable aspect of therapy, and in itself is associated with cognitive morbidity such as deficits in memory, processing speed, attention and executive functions,^102^ core skills supporting reading.^2^ Even patients with well-demarcated supratentorial tumors suffer measurable sequelae, including white matter structural disruption.^103^ Cranial radiotherapy is significantly associated with negative long-term cognitive impacts due to direct injury to neurons, glia, and vascular structures, moderated by age, dose, and radiation field. Although technical advances have abrogated some of these risks, the impact of CNS radiotherapy on cognitive function has been quantified as a decline in intelligence quotient (IQ) of 2–8 points per year of treatment.^104,105^ Quantifying the cognitive impact of additional factors such as chemotherapy-associated ototoxicity, anesthesia, endocrinopathies, and other host and environmental disease modifiers are a priority in brain tumor outcomes research.^106^

While cancer diagnosis and treatment during early childhood can confer substantial risks for reading difficulties, these can be mitigated by interventions.^107,108^ As interactions with multidisciplinary oncology teams tend to occur frequently, these afford opportunities for the provision of sustained reading and literacy resources, guidance, and surveillance, ideally initiated in a proactive manner throughout the course of therapy. Although the need is well-recognized, knowledge of assessment and intervention tools is highly variable. For example, in a survey of 282 physicians from Children’s Oncology Group (COG) institutions, pediatric oncologists nearly unanimously reported a primary responsibility to deliver information to families about the impact of cancer therapies on cognition (e.g., reading) and school, yet only 66% reported even moderate understanding of issues that their patients may face upon school re-integration. Furthermore, 54% of oncology providers reported not having received any training in reading- and school-related areas.^109^ However, beyond anticipatory guidance and patient education, early intervention in these areas has been cited as a priority by the COG survivorship committee.^110^ Consensus guidelines have identified areas of concern including child handwriting, memory, concentration, reading comprehension, vocabulary, and spelling, particularly in those receiving intrathecal therapy, CNS radiation or surgery, or platinum chemotherapeutics that may impact hearing.^110^ Mitigation strategies have also been outlined including referral to psychologists and other educational specialists, yet are dependent on parent, teacher, or school initiation.^110^ Tools are also available including home-based (e.g., computerized working memory intervention) and hospital-based (e.g., School Liaison Program) educational supports, which compliment school-based programs (e.g., Special Education Services) and have been associated with variable benefits.^111^

Given oft-lengthy and/or frequent hospital stays during treatment, inpatient programs have the potential to provide shared reading experiences, encouragement, academic continuity, and to address potential deficits for children with cancer diagnoses prior to discharge. This was affirmed in a 2016 review, which summarized the benefits of interventions designed to assist children with cancer in the transition back to school.^112^ While focused on psycho-social outcomes, it was clear that these can also be effective in terms of reading and literacy. Overall, there are major gaps and opportunities in need of research on inpatient and outpatient levels, including integration of reading-related measures into pediatric cancer clinical trials, which may help inform collaborative approaches to screening, prevention, and intervention.

#### Congenital heart disease

Congenital heart disease (CHD) is the most common birth defect, affecting approximately 9 in 1000 live births in the US.^113^ Approximately 25% of children with CHD require cardiac surgery during infancy.^114^ Common types of CHD requiring surgery in the first year of life include complex ventricular septal defects (VSD), pulmonary atresia, tetralogy of Fallot, hypoplastic left heart syndrome, and coarctation of the aorta.^115,116^ Surgical procedures for complex CHD require sedation and general anesthesia, potential contributors to well-documented neurodevelopmental delays in affected children.^117^ There are many other possible causes across the prenatal to postnatal spectrum, including genetics and epigenetics, altered fetal circulation, placental alterations, prematurity and low birth weight, poor nutrition, chronic cyanosis, neurological injury, and chronically high levels of parent and child psychological stress. Environmental factors such as maternal education can moderate reading outcomes in children with CHD, highlighting the importance of family context and support.^118^ Intraoperative factors during CHD surgery, such as cardiac bypass, have been found to contribute a smaller proportion of the variance in neurodevelopmental outcomes than previously thought.^119,120^ An in-depth examination of neurodevelopmental risks for children with CHD can be found in.^121,122^

There is a relatively large body of research investigating neurodevelopmental outcomes among children with CHD,^117,123^ including receptive and expressive language delays in both surgically and non-surgically treated groups.^124^ Children with CHD have been shown to perform poorly relative to peers on tests of language at all ages, including relevant domains of the Bayley Scales of Infant Development (BSID-III) in young children,^125^ Wide Range Achievement Test in older children (WRAT),^126^ grade-level reading achievement tests.^118,127^ The Cardiac Neurodevelopmental Outcome Collaborative (CNOC) recently published a comprehensive set of recommendations for neurodevelopmental evaluation in children with CHD, including the use of standardized, performance-based, multi-domain evaluations across the lifespan.^128,129^ Risks of delays are highest for children with more complex CHD, longer hospital stays, and/or comorbidities.^118,130,131^ As a result, children with complex CHD are up to 50% more likely to utilize special education services than children in the general community;^118,132^ however, while language and literacy are strongly linked,^133^ to our knowledge no studies have specifically examined longer-term reading and literacy outcomes for children with CHD.

Neurodevelopmental surveillance, screening, and evaluation, as well as transition planning, are areas of increasing focus within CHD care.^134,135^ This includes the use of age-appropriate modes of communication for very young children with CHD, such as gestures^125^ and joint attention,^133^ which can be predictive of longer-term language skills. While promotion of reading and literacy is not typically seen as a part of routine cardiac care and has not been specifically studied in the CHD population to date, cardiac neurodevelopmental clinics are an ideal location for their incorporation and study. This might include incorporation of reading into the individualized patient- and family-centered care plans,^136^ and providing reading and academic support during inpatient stays. Fortunately, neurodevelopmental clinics are becoming more prevalent since the publication of the American Heart Association (AHA) and American Academy of Pediatrics (AAP) statement on the need for ongoing developmental monitoring and assessment from infancy through to adolescence and beyond.^137^ Currently there are more than 50 centers with cardiac neurodevelopmental clinics across the United States and Canada (https://cardiacneuro.org/institutions/). The AHA/AAP scientific statement provides clear guidelines for how to risk-stratify patients with CHD to inform timely referral to services for evaluation and management;^137^ however, given the incidence of CHD and the low number of neurodevelopmental clinics, there is currently an imbalance in supply and demand for such services, highlighting the central role of primary cardiologists and pediatricians for surveillance and management. Current recommendations include early risk identification using validated instruments, and referral to Early Intervention services to mitigate existing, congenital risk.^128^ Research is needed to optimize how these might be implemented on outpatient and inpatient levels.

#### Epilepsy

Epilepsy is one of the most common neurological disorders diagnosed during childhood (~1 in 150 children by age 10), affecting ~0.5% to 1% of children worldwide.^138^ It is characterized by at least one unprovoked seizure per year, with the expectation that these are likely to recur.^139^ Epilepsy type is determined by seizure locus, extent (e.g., involving loss of consciousness) and frequency. The two most common types are benign childhood epilepsy with centrotemporal spikes (BCECTS), also known as Rolandic epilepsy, and temporal lobe epilepsy (TLE). While exact rates in children are unknown, TLE is most prevalent (~60% of cases), affects all ages, and tends to persist,^140^ while BCECTS (~15% of cases^141^) is most often diagnosed between ages 3 and 13 years and is often outgrown by age 18.^142,143^

Potential causal factors for cognitive delays in children with epilepsy include brain structural differences, ion channelopathies, aberrations in central nervous system apoptosis and/or synaptic function and damage during prolonged seizures (e.g., via metabolic acidosis).^144,145^ Alone or combined, these can impact maturation and/or connectivity of brain areas required for an efficient reading network (e.g., language, visual, attention).^21,146^ As these areas are widely distributed across the brain, it is not surprising that children with epilepsy tend to experience varying degrees of cognitive and reading impairment impacting school performance.^142,143,147–149^ Specific risk factors include earlier age of seizure onset and greater frequency, duration, and severity of seizures.^144,150,151^ Side effects of some anti-epileptic drugs (AEDs; e.g., phenobarbital, topiramate) can convey risks (e.g., cognitive slowing), though these are reduced with newer agents.^152^ Surgical procedures required to ablate intractable seizure foci can also impact cognitive abilities, depending on areas involved.^153,154^ An important distinction from other chronic conditions described in this review is that epilepsy management (assuming well-managed) rarely involves prolonged hospitalization or surgical procedures. Thus, reading difficulties in children with epilepsy is most often linked to direct disease and/or treatment factors, and less often to indirect factors such as missed school.

While some types of epilepsy are associated with mildly (e.g., BCECTS) to profoundly low IQ (e.g., infantile spasms),^144^ in most epileptic children IQ is normal (74%).^143,155,156^ However, even with normal intelligence, a sizable proportion of epileptic children experience cognitive difficulties.^156–158^ These include difficulties with attention (20–50%^159,160^) and executive function (30–50%^161,162^) notably working memory and speed of processing,^163^ which are critical to read fluently^164^ and support comprehension.^165^ As the temporal lobe (typically left-lateralized) is a core of both language and reading networks,^166^ TLE almost always involves foci with the potential to impair these abilities. While currently under-researched, there are significant links between TLE in children and longer-term reading (e.g., accuracy, comprehension) and academic difficulties.^167^ Reflecting the heterogeneity of deficits in epileptic children, BCECTS has been associated with difficulties in phonological processing, which is a core deficit in dyslexia.^144,168^ Indeed, long-term reading outcomes are significantly worse in dyslexic children with BCECTS compared to non-epileptics.^169^ Anxiety, depression, and attention-deficit hyperactivity disorder (ADHD) are also often comorbid with epilepsy, and can exacerbate reading and academic difficulties.^170,171^ Altogether, 13–17% of children with epilepsy experience reading difficulties^19,172^ and up to 50% more general learning issues.^149,173^

Given its centrality as academic demands shift from “learning to read, to reading to learn” (usually around 3rd grade), reading can be reasonably viewed as the root of wider academic struggles in epileptic children.^174–176^ As with other chronic medical conditions, parents and healthcare providers can play active roles to help mitigate risks for reading difficulties in epileptic children.^147,177^ While studies have suggested normalization of behavioral and academic performance upon resolution of seizures, ongoing surveillance, and support for epileptic children have been advocated.^178^ For those diagnosed with reading difficulties, interventions should be tailored to deficits related to underlying pathology (e.g., addressing phonological skills for children with BCECTS). There have been reports of improved cognitive abilities via computerized reading training in epileptic children, suggesting potential for such an approach.^179^ In terms of transitioning, while a resolution of seizures in some cases may render self-management less of a concern relative to other chronic conditions, in general children with higher literacy levels will be better equipped to manage all aspects of their health. However, no large-scale studies involving approaches to optimize reading outcomes for epileptic children have currently been published and further research is needed.^167^

#### Sickle cell disease

Sickle cell disease (SCD) is a heritable red blood cell (RBC) disorder involving a homozygous-recessive mutation of the hemoglobin-Beta gene on Chromosome 11, causing RBCs to have an abnormal “sickle” shape and impair circulation, particularly during times of stress or acute illness.^48,180^ Estimated incidence of SCD is ~1 in 365 Black/African American and 1 in 16,300 Hispanic infant births per year.^48^ Attributable to impaired circulation and tissue damage, complications of SCD include severe and chronic pain, infections, acute chest syndrome, and cerebrovascular accident (stroke).^48^ The latter is the most obvious and severe direct cause of cognitive impairment, while frequent school absence,^181^ under-resourced home literacy environment,^182^ and depressive, anxious, and inattentive symptoms linked to pain and stress of illness^183^ can all confer additional indirect risks.^184^ As marginalized populations, Blacks and Hispanics bear outsized risks for inequities in HLE and literacy-supporting resources (e.g., access to quality early childhood education,^185,186^) exacerbating adverse outcomes.^187,188^

While a diagnosis of SCD does not guarantee that a child will experience learning difficulties, associated comorbidities place children at outsized risk.^188,189^ Fortunately, routine newborn screening, and proactive interventions have significantly decreased stroke in pediatric SCD populations (overall incidence in affected children ~10%), which has been a major risk factor.^190,191^ Silent cerebral infarct (SCI), however, is relatively more common (17–30%),^190–192^ conveying more subtle risks to components of the reading network and abilities.^21^ Evidencing this, studies affirm that children with SCD and no stroke history often experience impairments in memory, language, and executive functions,^191^ core skills supporting reading and literacy. Deficits in executive function have been described in preschool-aged children with SCD,^193,194^ and in language abilities in 5- to 7-year-old children, attributed to impaired cerebral blood flow.^195,196^ While the evidence is limited, such deficits may, in turn, underlie lower kindergarten readiness and longer-term risks of reading difficulties in children with SCD.^197^ For example, a study of adolescents with SCD found that ~40% received special education services, and a majority frequently missed school due to illness (an indirect risk factor).^181^ Indeed, neuropsychological test scores and academic performance in children with SCD tend to worsen with age, though the degree of impairment is variable.^191,198^ A longer-term consequence, however, is arrested academic and vocational achievement (e.g., not graduating from high school or attending college), which is well-established.^181,199,200^

Despite substantial risks for children with SCD, factors like parental education and HLE play a significant role in moderating outcomes.^201^ Thus, engaging parents not only in the management of physical and psycho-social aspects of care, but also in the importance of nurturing emergent literacy abilities and interest in reading (e.g., via consistent shared reading routines) beginning at a young age are critical.^202^ Comprehensive developmental screening in children with SCD could enable stratification by risk and resilience (e.g., family and neighborhood resources) levels for reading and subsequent academic difficulties.^203,204^ Qualitative studies have provided insights into how academic impacts of SCD might be mitigated, including clear communication with teachers and administrators at all ages, advocacy to raise awareness of challenges faced by children with SCD, and accommodations as needed.^205^ However, despite parental concerns and eligibility, other studies have found the limited receipt of services (e.g., Section 504 accommodation plans) for children with SCD.^206^ Further, there is currently limited research surrounding early interventions targeting emergent literacy and reading in children with SCD. Similarly, research involving literacy and its relationship to transitioning to adult care in adolescents with SCD is limited.^207,208^ This is concerning, as studies have found that health literacy in adolescents with SCD tends to be low,^181,209^ predicted by earlier cognitive abilities^210^ and independent of caregiver health literacy.^211^ This is compounded by evidence that educational materials intended for parents of children with SCD may exceed usual literacy levels.^212^ Combined, these factors are likely to impair the transitioning process and subsequent academic, social, and health outcomes for this underserved population.^213,214^

## Discussion

Literacy is a major social determinant of health and is linked to numerous outcomes of interest to pediatric providers, educators, advocates, and policymakers.^215,216^ It is amenable to an eco-bio-developmental model accounting for the range of factors influencing those impacting the integration of an efficient, functional reading brain network, and consequent targets for interventions.^3^ As summarized here, asthma, cancer, CHD, epilepsy, and SCD are chronic conditions affecting millions of children in the US and worldwide. Each of these convey substantial risks of reading difficulties via direct disease effects on the brain and indirect effects on child, family, and school routines. Care for children with these and other conditions involves cooperation among pediatric primary care and specialty providers, therapists, psychologists, educators, and families, with a unified focus on optimizing neurodevelopmental outcomes beginning as early as possible. Integration of timely screening, inclusion of reading and literacy risks and difficulties in care management plans, implementation of evidence-based HLE enrichment programs,^36,217^ and therapies targeted to both core (e.g., language) and supporting (e.g., attention) domains offer promising synergies, leveraging access by trusted primary and specialty care teams during formative stages of development.

Pediatric primary care and specialty providers may be less aware of the risks of reading difficulties in children with chronic illness, relative to medical concerns that are often complex and even life-threatening. Medical problem lists tend to be the focus of clinic visits, and families may reasonably focus questions on the child’s physical and psycho-social health, particularly at young ages.^218,219^ While this is evolving, reading and literacy have tended to be viewed as a “school issue,” beyond the purview of usual pediatric training and practice and outside the view of what is typically addressed during a clinical encounter.^220^ Evidence featured in this review highlights the importance of incorporating reading-related risks into problem lists and care management plans for children with chronic illness (e.g., need for language testing, potential side effects of AEDs), also considering demographic factors likely to amplify these risks (e.g., parental education, HLE). The potential impact of comorbid mental health issues and broader cognitive delays on reading and literacy outcomes should also be noted.^221^

**Fig. 1:**
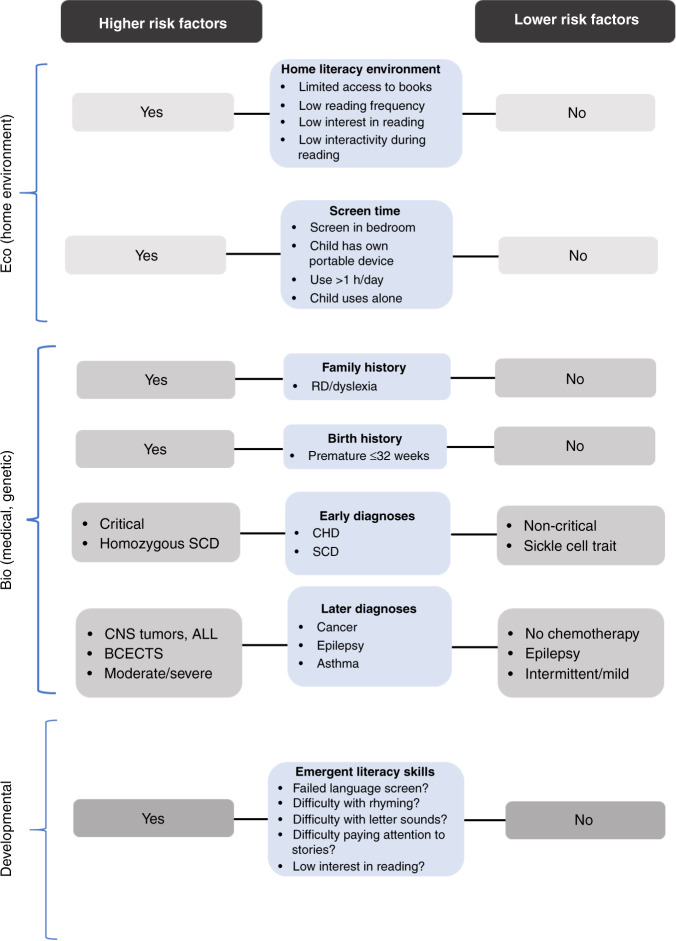
Risk stratification for eco, bio, and developmental factors influencing emergent literacy development in children with chronic and complex illness. Medical conditions can convey both direct (bio) and indirect (eco) risks. Potential “red flags” are suggested: (1) failed language screen or documented delay prior to age 5, (2) difficulty rhyming by kindergarten, (3) difficulty matching letters with their sounds between grades 1–3, (4) Difficulty paying attention to stories at and after age 3, (5) low interest in reading in grade 3-up.

**Fig. 2:**
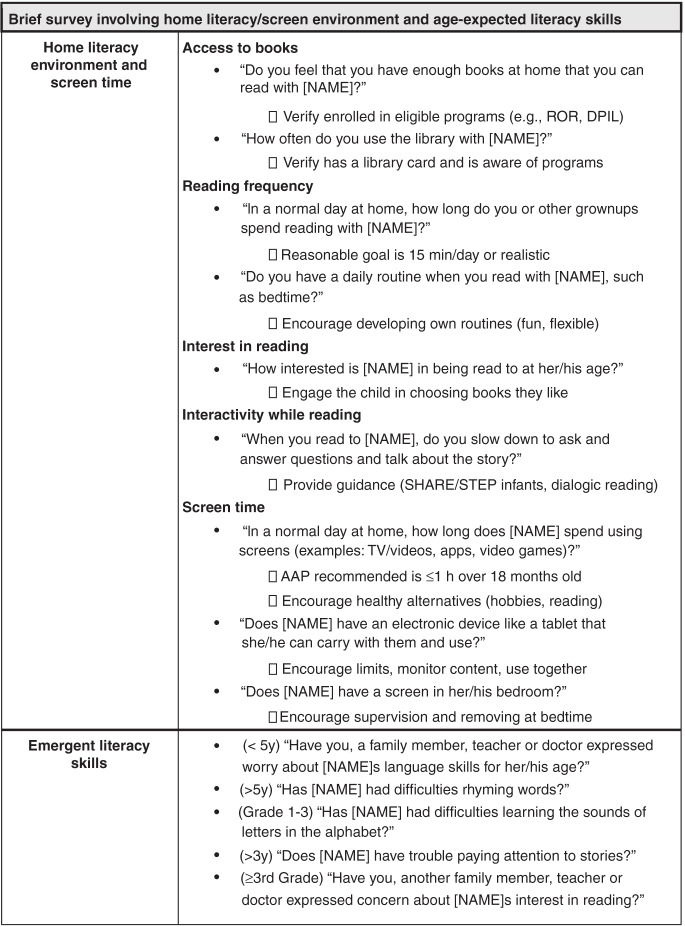
Brief screening questionnaire for pediatric providers to help identify potential risks for reading difficulties. These include modifiable behaviors in the home (reading and screen-based media use) and parental concerns regarding the child’s emergent literacy skills (age 3 and older). Items are adapted from AAP literacy^13^ and screen-based media^24^ recommendations and a review of typical, evidence-based reading milestones.^2^

While validated tools are available to detect signs of potential reading difficulties as young as age three,^35,222^ literacy screening is currently not routinely performed in general or specialty practice. Similarly, brief screening measures for older children exhibiting signs of reading difficulties have been developed,^223^ yet are not widely used. Holistic assessment of HLE and demographic factors likely to impact reading and literacy development (e.g., need for adult literacy services and/or help to access quality preschool) would also be worthwhile, yet this is not a typical approach. A major reason may be that despite AAP recommendations,^13^ literacy promotion is not currently reimbursed by insurance (though developmental screening is), presenting providers with a seemingly impossible task given time constraints and a range of developmental and/or safety topics to cover. This seems a missed opportunity, as primary care and specialty providers are poised to administer reading and literacy surveillance, guidance, and interventions beginning at a young age. A succinct diagram to help stratify risk is provided in Fig. 1, and a brief survey involving potential risks adapted from AAP guidelines and “red flags” at various ages relative to typical milestones is provided in Fig. 2.^2^ While not featured here, succinct health literacy screening measures are also available to help frame care and transitioning guidance (e.g., REALM,^224^ Newest Vital Sign.^225^)

Existing literacy promotion programs based in pediatric practice focus on enhancing HLE prior to kindergarten, especially for families from impoverished neighborhoods.^36,217^ The most established is ROR (endorsed by the AAP), which reaches ~25% of U.S. children in poverty via a network of over 33,000 primary care clinicians who provide a new book and guidance at well-visits from infancy to 5 years old.^36^ The ROR evidence base includes enhanced shared reading attitudes and routines, child language, kindergarten readiness,^226^ and satisfaction with clinic visits.^227^ Clinic participation in the ROR program has also been associated with higher attendance at well-visits, particularly among lower-educated and minority families.^228^ Reasons include higher staff morale, positive provider-family interactions, and value assigned to clinic visits by families.^229^ While links with particular diagnoses have not yet been studied, it is reasonable to infer that disease-specific benefits reasonably accrue from higher attendance and engagement at well-visits, where management of chronic illness is a focus. It is also reasonable to infer that similar benefits may manifest during specialty care, though at present, there are no similar programs based in specialty clinics.

Reading programs in Newborn Intensive Care Units (NICUs) have recently shown promise in terms of reduced family stress, improved reading attitudes, and enhanced staff morale compared to usual practice.^37,38^ However, no tested, reading-centric programs currently exist in pediatric inpatient care, reflecting opportunities for innovation and research. For example, tech-enabled approaches (e.g., mobile apps), which are widely used for pediatric health and disease management on inpatient and outpatient levels,^230^ have shown promise to enhance literacy promotion during primary care.^231^ Given privileged and often frequent access to families by trusted medical teams, including in clinics focused on neurodevelopment, there is potential for incorporation of such value-added tools for children with medical complexity, as featured in this review. Further, with over 2-million pediatric hospitalizations in the U.S. annually,^232^ many involving prolonged stays for chronic conditions,^233^ inpatient care seems opportune for enhanced exposure to reading and literacy materials and guidance.

While beyond the scope of this review, it is vital to note the intersection of general and health literacy for children and caregivers,^9^ which impact comprehension of educational materials, adherence, and outcomes at all ages.^234–238^ Children with reading difficulties are especially vulnerable to a lower understanding of their health condition, rationale for therapies, and treatment plans, which is likely to worsen with age as more is expected of them, particularly if literacy in family members is also low. This scenario is likely to impact the vital process of transitioning to self-care in adolescence and into adulthood. Thus, optimizing emergent literacy trajectories and reading abilities beginning in early childhood can provide a foundation for enhanced health literacy and reduced morbidity, mortality, and healthcare costs. This applies not only to the five conditions discussed in this review, but potentially to other complex and/or chronic pediatric conditions.

Compared to genetic and environmental risk factors for reading difficulties, scant research has been conducted to characterize risks for children with many complex and chronic health conditions, particularly those not clearly linked with developmental delays. This includes more obvious direct (e.g., vascular insults in SCD, temporal epileptic foci, intrathecal chemotherapy) and indirect (e.g., school absenteeism) risks, more subtle risks (e.g., stress of illness, displacement of reading routines), and those that apply for all children (e.g., impoverished HLE). Similarly, the intersection of general and health literacy in the context of medical complexity is currently under-studied, impacting treatment and transitioning strategies.^238^ It is clear from this review that longitudinal research is needed applying an eco-bio-developmental model where emergent literacy is framed as a distinct developmental domain reliant on efficient integration of the functional reading network.^239^ This has the potential to align primary and specialty providers with educators and families across a range of conditions,^222^ to optimize strategies and outcomes.
